# Intravenous Infusion of Mesenchymal Stem Cells Enhances Long-Tract Reorganization That Circumvents the Lesion in a Rat Staggered Hemisection Spinal Cord Injury Model

**DOI:** 10.3390/biomedicines14091979

**Published:** 2026-09-02

**Authors:** Hisashi Obara, Masahito Nakazaki, Takahiro Yokoyama, Hiroshi Nagahama, Ryunosuke Fukushi, Shinichi Oka, Ryo Ukai, Yuko Kataoka-Sasaki, Masanori Sasaki, Jeffery D. Kocsis, Atsushi Teramoto, Toshihiko Yamashita, Osamu Honmou

**Affiliations:** 1 Department of Neural Regenerative Medicine, Institute of Regenerative Medicine, Sapporo Medical University School of Medicine, Sapporo 060-8556, Hokkaido, Japan; h.obara19870927@sapmed.ac.jp (H.O.); takahiro_yokoyama@sapmed.ac.jp (T.Y.); h.nagahama@sapmed.ac.jp (H.N.); ryunosuke@sapmed.ac.jp (R.F.); soka@sapmed.ac.jp (S.O.); ryou@sapmed.ac.jp (R.U.); yuko.k.sasaki@sapmed.ac.jp (Y.K.-S.); msasaki@sapmed.ac.jp (M.S.); honmou@sapmed.ac.jp (O.H.); 2 Department of Orthopedic Surgery, Sapporo Medical University School of Medicine, Sapporo 060-8556, Hokkaido, Japan; atsushit@sapmed.ac.jp; 3 Department of Neurology, Yale University School of Medicine, New Haven, CT 06510, USA; jeffery.kocsis@yale.edu; 4 Division of Radioisotope Research, Biomedical Research, Education and Instrumentation Center, Sapporo Medical University School of Medicine, Sapporo 060-8556, Hokkaido, Japan; 5 Division of Neuroscience, Department of Physiology, Sapporo Medical University School of Medicine, Sapporo 060-8556, Hokkaido, Japan; 6 Department of Neuroscience, Yale University School of Medicine, New Haven, CT 06510, USA; 7 Center for Neuroscience and Regeneration Research, VA Connecticut Healthcare System, West Haven, CT 06516, USA; 8 Sapporo Medical University, Sapporo 060-8556, Hokkaido, Japan; tyamasit@sapmed.ac.jp

**Keywords:** mesenchymal stem cells, spinal cord injury, diffusion tensor imaging, axonal plasticity, neural tract reorganization

## Abstract

**Background/Objectives**: Non-contiguous dual spinal cord injuries are associated with poor neurological outcomes, probably due to reduced spared-tract bridges for compensatory reorganization. Intravenous (IV) infusion of mesenchymal stem cells (MSCs) promotes recovery after SCI. However, how MSCs influence long-tract reorganization across multiple lesions remains unclear. We characterized MSC-induced long-tract plasticity at two separate lesion levels using ex vivo diffusion tensor imaging (DTI) in a rat staggered hemisection model. **Methods**: Adult rats underwent staggered lateral hemisection at right T7 and left T12 and received IV MSCs (1 × 10^6^) or vehicle on day 1; locomotor recovery was assessed over 4 weeks (using Basso–Beattie–Bresnahan (BBB) scale) and ex vivo DTI tractography was performed at 4 weeks (7.0 T). **Results**: MSC-treated rats showed significantly greater locomotor recovery than vehicle-treated rats (BBB at day 28:12.2 ± 0.6 vs. 5.4 ± 0.7, *p* < 0.001). MSC infusion increased total, preserved, and redirected tract counts at both lesion levels; at T7, redirected tracts entered the non-injured lateral funiculus, forming detour pathways bypassing the lesion, whereas at T12, redirected tracts from the injured-side dorsal funiculus crossed the midline into the contralateral dorsal funiculus. **Conclusions**: Intravenous infusion of MSCs improved locomotor outcome and altered the level-specific patterns of long tracts reconstructed from DTI data. Ex vivo DTI provides an overview of these structural changes.

## 1. Introduction

Spinal cord injury (SCI) involving multiple spatially separated lesions, particularly non-contiguous dual spinal cord injuries (NDSI), is a severe form of spinal trauma. A large proportion of patients within this population present with complete motor and sensory loss (ASIA grade A) and show limited neurological recovery [1,2]. Functional recovery after SCI depends largely on spared tract bridges that serve as critical substrates for axonal reorganization [3,4]; a second non-contiguous lesion can potentially further deplete this reserve and constrain the compensatory reorganization that normally supports recovery after a single-level injury. However, treatment strategies that specifically address the unique challenges posed by dual or multi-lesion SCI remain lacking.

Intravenous (IV) infusion of bone marrow mesenchymal stem cells (MSCs) has emerged as a promising therapeutic strategy for SCI [5]. One therapeutic mechanism involves enhancing neural or axonal plasticity. Accumulating experimental evidence indicates that IV-MSC therapy amplifies plastic responses within spared long descending systems, promoting interactions among multiple motor pathways and reinforcing compensatory connectivity below the lesion [6,7]. In particular, IV-MSC infusion enhances bridging connections not only within the corticospinal tract (CST) via enhancing fine-caliber pre-existing axons (increased axonal diameter) [6], but also in extrapyramidal descending pathways such as the rubrospinal tract (RST), through increasing axonal signal intensity [7]. Therefore, MSC-based interventions offer a rational approach for restoring latent plasticity and may improve outcomes, even for NDSI.

Ex vivo diffusion tensor imaging (DTI) is a powerful tool to clarify the modifications in neural plasticity, particularly within the long descending tracts, under these conditions. DTI enables unbiased reconstruction of axonal trajectories throughout the entire spinal cord, in contrast to conventional tract-tracing techniques, which are limited to selected neuronal populations [8]. By systematically adjusting the region of interest (ROI) placement and inter-ROI distance, the ex vivo DTI approach enables evaluation of neural tracts independent of their anatomical location or trajectory within the spinal cord, and allows preferential weightage of the analysis toward long-projecting pathways while minimizing contributions from interneuron-mediated neural network reorganization [9,10].

In this study, we performed a comparative ex vivo DTI–based analysis of axonal tract reorganization in a staggered lateral hemisection SCI model [11], which is an experimental analog of NDSI. Staggered hemisections were introduced at the T7 and T12 levels, followed by intravenous infusion of MSCs. This model allows DTI to systematically evaluate MSC-induced structural reorganization across both lesion sites by assessing axonal trajectories within the rostral, lesion, and caudal segments at each level, thereby enabling the identification of MSC-induced long-tract remodeling patterns.

## 2. Results

### 2.1. Intravenous MSC Infusion Improves Locomotor Recovery After SCI

**Figure 1:**
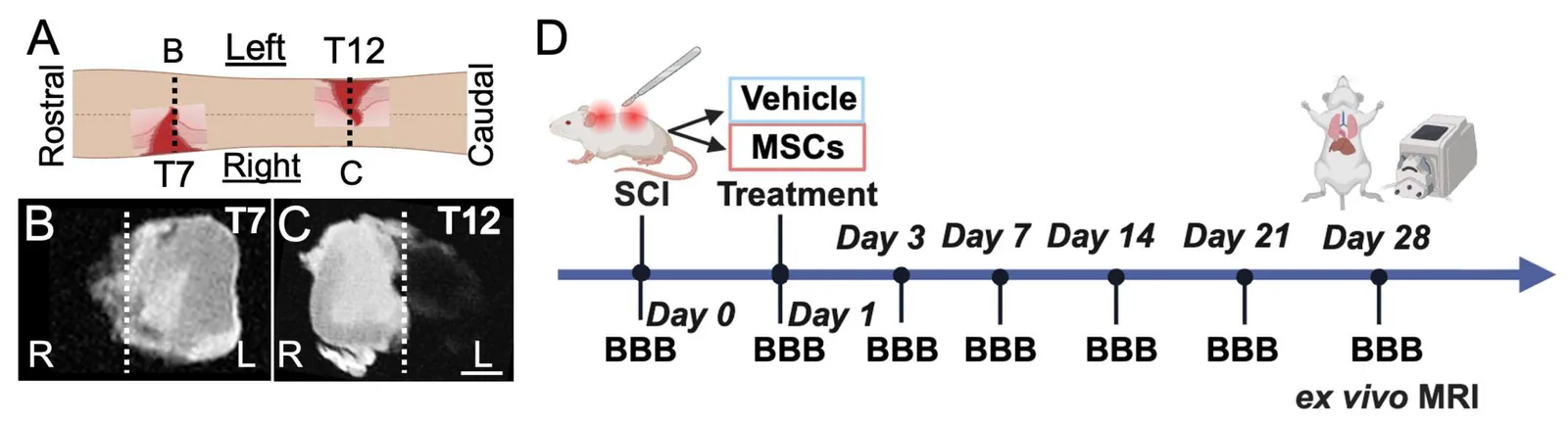
Experiment protocol (**A**) Schematic illustration of the staggered lateral hemisection model showing a right-sided lesion at T7 and a left-sided lesion at T12. Corresponding axial T2-weighted MRI images confirm the lesion locations at the T7 (**B**) and T12 (**C**) levels. (**D**) Experimental timeline. Rats received either vehicle or MSC infusion via the femoral vein on day 1 after SCI. Locomotor function was evaluated using the BBB scoring scale on days 0, 1, 3, 7, 14, 21, and 28. Samples for ex vivo MRI were collected on day 28. MSCs = mesenchymal stem cells, BBB = Basso–Beattie–Bresnahan locomotor scale score, SCI = spinal cord injury, MRI = magnetic resonance injury.

To recapitulate a spatially staggered, discomplete SCI, rats underwent a staggered lateral hemisection consisting of a right-sided lesion at T7 and a left-sided lesion at T12 (Figure 1A); the location of each hemilesion was confirmed on axial MRI at the T7 and T12 levels (Figure 1B,C). One day after SCI, the rats received an IV infusion of either vehicle or MSCs via the femoral vein, and locomotor recovery was monitored for 28 days (Figure 1D).

**Figure 2:**
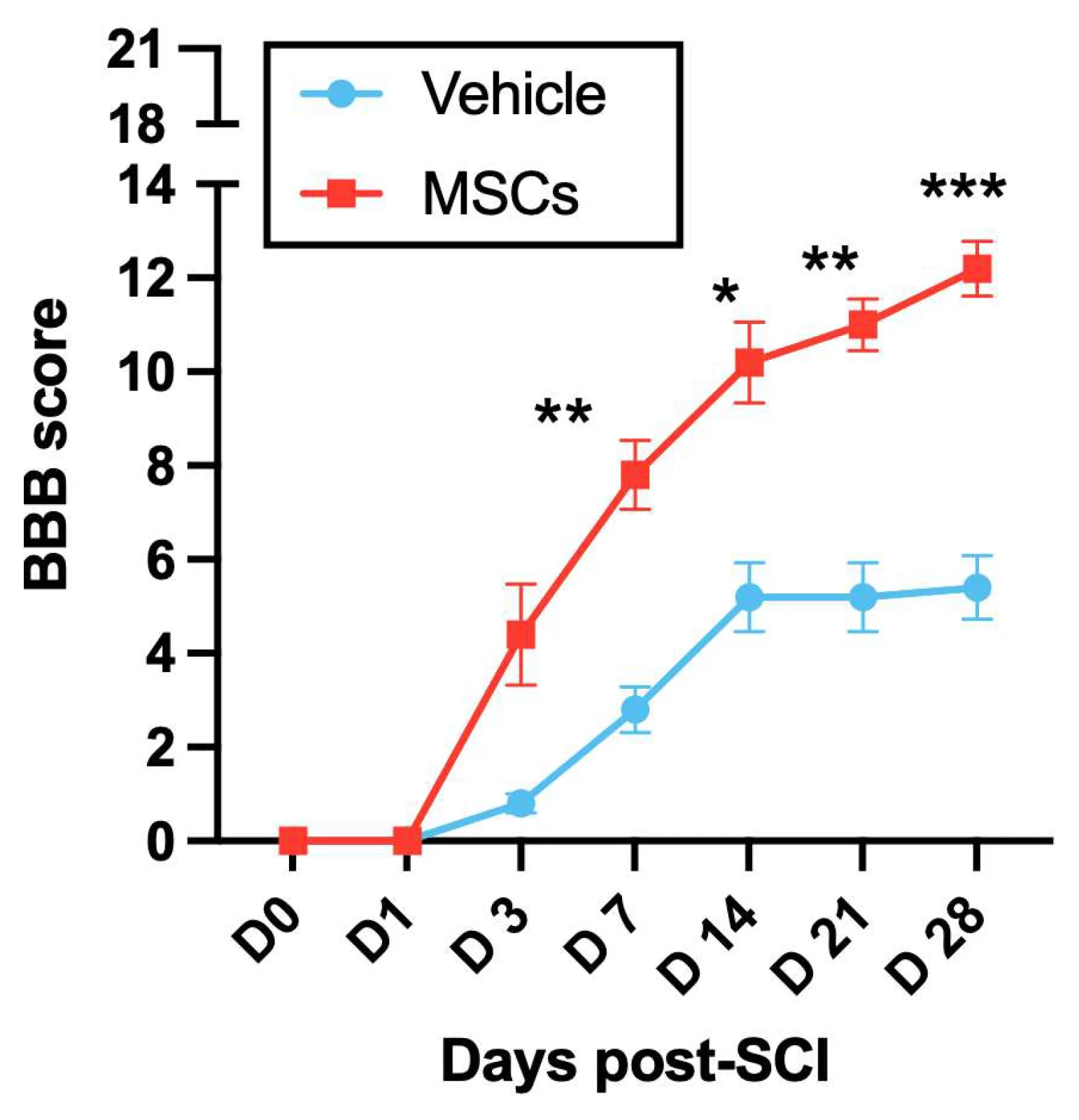
Intravenous MSC infusion promotes locomotor recovery following staggered lateral hemisection SCI. Open-field locomotor scores. BBB scores were measured from day 0 to day 28 after SCI. Both groups displayed complete paraplegia on day 1. Vehicle-treated rats showed only limited recovery, whereas MSC-treated rats exhibited progressive improvement. Significant differences emerged from day 7 onward. Data are presented as mean ± SEM; Vehicle (*n* = 5), MSC (*n* = 5). Intact rats maintained a score of 21. Statistical analysis: repeated-measures ANOVA with Šidák’s post hoc test. * *p* < 0.05, ** *p* < 0.01, *** *p* < 0.001. MSCs = mesenchymal stem cells, BBB = Basso–Beattie–Bresnahan locomotor scale, SEM = standard error of mean, ANOVA = analysis of variance.

Open-field locomotor function was assessed using the Basso–Beattie–Bresnahan (BBB) scoring scale on days 0, 1, 3, 7, 14, 21, and 28 after SCI (Figure 1D). On day 1 post-SCI, all rats in both groups showed complete paraplegia (BBB score of 0). Open-field locomotor testing showed that intravenous MSC infusion significantly improved locomotor recovery after severe SCI (Figure 2). Vehicle-treated rats exhibited only minimal spontaneous recovery, with BBB scores plateauing at 5.4 ± 0.7 by day 28. In contrast, MSC-treated rats demonstrated progressive recovery, reaching 12.2 ± 0.6 at day 28 with sustained improvement. Although the difference on day 3 did not reach statistical significance (4.4 ± 1.1 vs. 0.8 ± 0.2), a significant difference between the groups was first observed on day 7, when MSC-treated rats achieved higher BBB scores (7.8 ± 0.7) than vehicle-treated rats (2.8 ± 0.5, *p* < 0.01). The effect was maintained at subsequent time points on day 14 (10.2 ± 0.9 vs. 5.2 ± 0.7, *p* < 0.05), day 21 (11.0 ± 0.5 vs. 5.2 ± 0.7, *p* < 0.01), and day 28 (12.2 ± 0.6 vs. 5.4 ± 0.7, *p* < 0.001). Collectively, these findings demonstrate that IV MSC infusion leads to a marked and sustained improvement in locomotor function compared with vehicle treatment in the staggered lateral hemisection SCI model.

### 2.2. MSC Infusion Promotes Tract Redirection and Preservation After Staggered Lateral Hemisection SCI at T7

**Figure 3:**
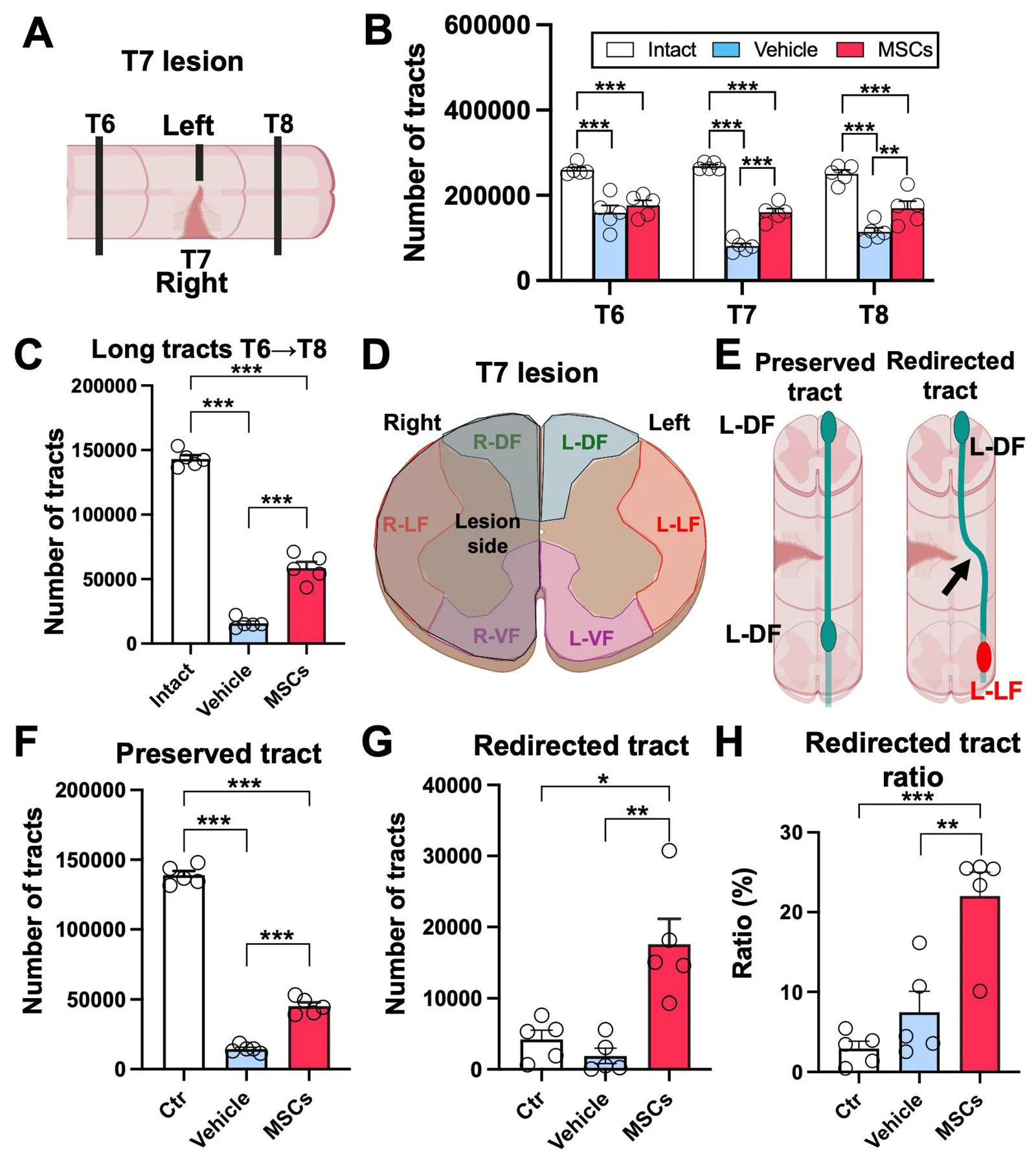
DTI tractography shows MSC-associated redirection and preservation of reconstructed tracts following staggered lateral hemisection SCI at T7. (**A**) Lesion schematic. Illustration of a staggered lateral hemisection lesion at T7 showing right-sided lesion. Black lines indicate the spinal levels (T6, T7, T8) analyzed using ex vivo DTI. (**B**) Total tract counts. Quantification of tract numbers at T6, T7, and T8 segments. (**C**) Long tract continuity. Quantification of longitudinal tracts spanning from T6 to T8. (**D**) Tract classification. Schematic of spinal cord cross-section at the T7 lesion level indicating the six ROIs used for tract categorization: R-DF, L-DF, R-LF, L-LF, R-VF, and L-VF. The lesion side (**right**) is indicated. (**E**) Definition of preserved vs. redirected tracts. Preserved tracts were defined as reconstructed tracts maintaining the same trajectory between T6 and T8 (e.g., L-DF to L-DF; **left panel**), and redirected tracts were defined as reconstructed tracts entering a different region at T8 compared to T6 (e.g., L-DF to L-LF; **right panel**). Arrows indicate the trajectory of reconstructed tracts from T6 to T8. (**F**) Preserved tracts. (**G**) Redirected tracts. (**H**) Redirected tract ratio. Data are presented as mean ± SEM. Group sizes: Intact (*n* = 5), Vehicle (*n* = 5), MSC (*n* = 5). Statistical analysis: one-way ANOVA with Tukey’s multiple comparisons test. * *p* < 0.05, ** *p* < 0.01, *** *p* < 0.001. DTI = diffusion tensor imaging, MSC = mesenchymal stem cell, SCI = spinal cord injury, ROI = region of interest, DF = dorsal funiculus, LF = lateral funiculus, VF = ventral funiculus, SEM = standard error of the mean, ANOVA = analysis of variance, R = right; L = left.

To assess whether intravenous MSC infusion altered reconstructed tract trajectories across the lesion, DTI tractography was performed in a staggered lateral hemisection SCI model. The analysis first focused on the T7 lesion level (Figure 3A). ROIs were placed to include the entire spinal cord cross sections at T6, T7, and T8. All tracts traversing each ROI were reconstructed and quantified to evaluate the total tracts at each segmental level. Compared with the intact group, the vehicle-treated SCI group had significantly reduced total tracts at the T7 (80,800 ± 6326 vs. 268,468 ± 3815; *p* < 0.001) and T8 levels (114,032 ± 9484 vs. 250,641 ± 9228; *p* < 0.001) (Figure 3B), as well as at the T6 level (159,019 ± 17,045 vs. 259,615 ± 5774; *p* < 0.001), despite this region being located rostral to the lesion site. In the MSC-treated group, total tract numbers at the T7 lesion core were reduced compared with the intact group (160,026 ± 9226; *p* < 0.001 vs. intact) but were significantly higher than those in the vehicle-treated SCI group (*p* < 0.001 vs. vehicle). At the caudal T8 level, tract numbers were significantly increased in the MSC-treated group (169,907 ± 16,758; *p* < 0.01 vs. vehicle) compared with the vehicle-treated group, although they remained lower than those in intact rats (*p* < 0.001 vs. intact). At the rostral T6 level, tract counts in the MSC-treated group (176,624 ± 11,569) remained lower than those in the intact group (259,615 ± 5774; *p* < 0.001) and were not significantly different from those in the vehicle-treated group (159,019 ± 17,045) (Figure 3B).

Subsequently, the longitudinal tracts between T6 and T8 were examined (Figure 3C). ROIs were placed at both T6 and T8 and the number of fiber tracts traversing both ROIs was quantified to evaluate long-tract continuity across the lesion. The analysis revealed that the total longitudinal tract numbers between T6 and T8 were markedly reduced in vehicle-treated rats compared with intact controls (15,823 ± 1706 vs. 143,150 ± 2876; *p* < 0.001). Tract numbers in the MSC-treated group were also lower than those in the intact group (58,615 ± 4801 vs. 143,150 ± 2876; *p* < 0.001). However, MSC infusion significantly mitigated tract loss compared with the vehicle-treated group (58,615 ± 4801 vs. 15,823 ± 1706; *p* < 0.001), indicating that MSC treatment attenuated the loss of reconstructable long-tract continuity across the lesion, although counts remained far below intact values (Figure 3C).

Next, we analyzed the trajectory patterns of the longitudinal tracts between T6 and T8. As illustrated in Figure 3D, six ROIs were placed across the cross-sectional area of the spinal cord at T6 and T8, corresponding to the left and right dorsal funiculi (DF), lateral funiculi (LF), and ventral funiculi (VF). In total, six ROIs were defined at the T6 and T8 levels, yielding 36 possible tract trajectories. Each tract passing through a given ROI at T6 was traced to determine which of the six ROIs it entered at T8. All 36 possible tract trajectories derived from the 6 (T6) × 6 (T8) ROI combinations were quantitatively analyzed and classified into two categories, “Preserved tract” and “Redirected tract.” Preserved tracts were defined as reconstructed tracts that maintained the same trajectory between T6 and T8, for example, those running through the left DF at both levels (shown in Figure 3E (left)). All other tracts that passed through different ROIs at T8 compared with their origin at T6 were classified as redirected tracts. These categories are operational descriptors based on ROI passage at the rostral and caudal levels (Section 4.5); a redirected tract indicates a change in the reconstructed trajectory and is not confirmed by histological analyses.

Quantitative analysis revealed markedly reduced preserved tracts in the vehicle group compared with the intact controls (14,498 ± 1189 vs. 138,923 ± 3026; *p* < 0.001), indicating extensive loss of preserved tracts after SCI (Figure 3F). In the MSC-treated group, the number of preserved tracts remained lower than that in the intact controls (45,241 ± 2618 vs. 138,923 ± 3026; *p* < 0.001) but was significantly higher than that in the vehicle group (*p* < 0.001), demonstrating partial protection of the preserved tracts across the T7 lesion (Figure 3F). The redirected tracts also showed marked differences between the groups. The number of redirected tracts was slightly lower in the vehicle group than in intact controls (1898 ± 1073 vs. 4227 ± 1280), whereas MSC infusion markedly increased redirected tracts (17,572 ± 3588), exceeding the values in both vehicle (*p* < 0.01) and intact groups (*p* < 0.01; Figure 3G). Moreover, the redirected tract ratio, defined as the percentage of redirected tracts relative to the total number of tracts (redirected/total × 100; Figure 3H), showed a slight but nonsignificant increase in the vehicle group compared with intact controls (7.5 ± 2.6% vs. 3.0 ± 0.9%). In contrast, this ratio was significantly elevated in the MSC-treated group (22.0 ± 3.0%), compared with both the vehicle (*p* < 0.01) and intact groups (*p* < 0.001), which indicates a greater number of tracts with altered trajectories across the T7 lesion. Collectively, these findings indicate that intravenous MSC infusion preserves the longitudinal tracts traversing the lesion and is associated with a higher number of redirected tracts, consistent with a higher number of tracts traversing the T7 lesion, both along preserved trajectories and along redirected ones.

### 2.3. MSC Infusion Preferentially Promotes Specific Redirected Tract Pathways at T7

**Figure 4:**
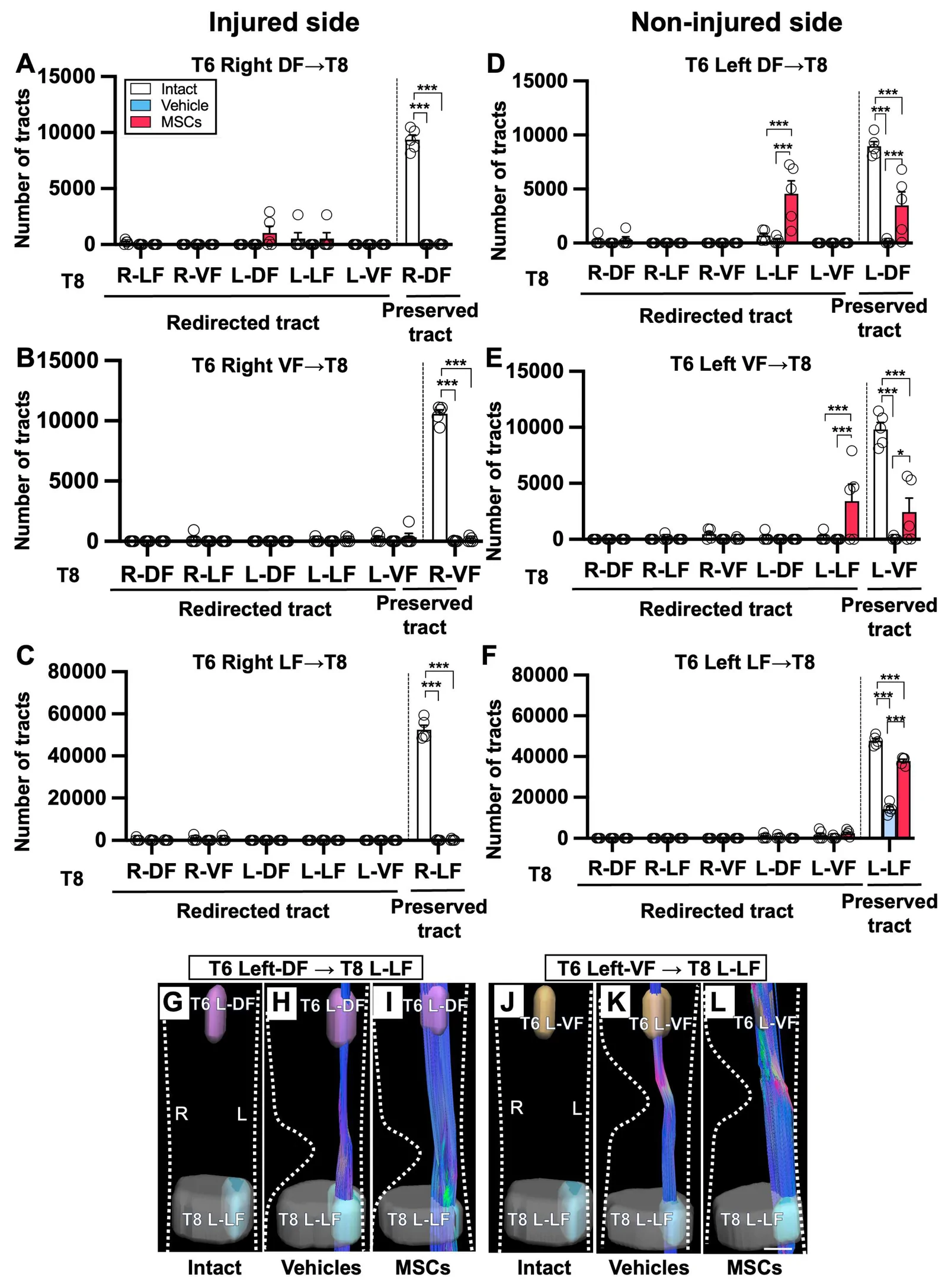
MSC infusion promotes selective redirection of axonal tracts across spinal pathways at the T7 lesion level. (**A**–**C**) Quantification of tracts originating from the injured (**right**) side at T6. (**A**) Right DF at T6 (R-DF → T8). (**B**) Right VF at T6 (R-VF → T8). (**C**) Right LF at T6 (R-LF → T8). (**D**–**F**) Quantification of tracts originating from the non-injured (**left**) side at T6. (**D**) Left DF at T6 (L-DF → T8). (**E**) Left VF at T6 (L-VF → T8). (**F**) Left LF at T6 (L-LF → T8). (**G**–**I**) Representative DTI tractography images showing redirected tracts from T6 L-DF to T8 L-LF. (**G**) Intact controls show minimal redirection. (**H**) Vehicle-treated rats display sparse redirected tracts. (**I**) MSC-treated rats exhibit markedly increased L-DF to L-LF redirection. (**J**–**L**) Representative DTI tractography images showing redirected fibers from T6 L-VF to T8 L-LF. (**J**) Intact controls show no evident redirection. (**K**) Vehicle-treated rats display minimal redirected fibers. (**L**) MSC-treated rats exhibit pronounced L-VF to L-LF redirection. Scale bar = 500 μm. Data are presented as mean ± SEM. Group sizes: Intact (*n* = 5), Vehicle (*n* = 5), MSC (*n* = 5). Statistical analysis: one-way ANOVA with Tukey’s multiple comparisons test. * *p* < 0.05, *** *p* < 0.001. DTI = diffusion tensor imaging; MSC = mesenchymal stem cell; DF = dorsal funiculus; LF = lateral funiculus; VF = ventral funiculus; SEM = standard error of the mean; R = right; L = left.

To identify which tracts passing through specific ROIs were preserved and which underwent MSC-induced redirection around the T7 lesion, we quantitatively analyzed the longitudinal tracts passing through each of the six ROIs defined at T6 (Figure 3D) to determine the six ROIs at T8 they projected to, using DTI tractography. Tracts running through the lesioned (right) side, including the right DF, VF, and LF at T6, are shown in Figure 4A–C, whereas those running through the non-injured (left) side are shown in Figure 4D–F. Here, the “injured” (lesioned) side refers to the side hemisected at the level analyzed and the “non-injured” side to the opposite side. Preserved tracts passing through the DF (Figure 4A), VF (Figure 4B), and LF (Figure 4C) on the T6 injury side were almost completely lost in the vehicle group compared with the intact controls, indicating severe disruption of longitudinal connectivity within the dorsal, ventral, and lateral pathways on the injured side. Even on the non-injured (left) side, preserved tracts were markedly reduced in the vehicle group compared with the intact controls (left DF (Figure 4D): Vehicle, 90 ± 86; Intact, 8964 ± 434; *p* < 0.001; left VF (Figure 4E): Vehicle, 73 ± 70; Intact, 9801 ± 635; *p* < 0.01; left LF (Figure 4F): Vehicle, 14,246 ± 1173; Intact, 47,743 ± 1079; *p* < 0.001). These findings suggest that progressive axonal loss extended beyond the lesioned side of the spinal cord, affecting the funiculi adjacent to the lesion (DF and VF) as well as the most distant region, the LF, on the non-injured side, which exhibited substantial tract loss.

In contrast, significantly reduced preserved tracts were observed on the lesioned side (DF, VF, and LF; Figure 4A–C) in the MSC-treated group compared with intact controls; however, on the non-injured side (DF, VF, and LF; Figure 4D–F), tract numbers were significantly higher than that in the vehicle group, but lower than that in intact controls (left DF (Figure 4D): MSC, 3495 ± 1246; *p* < 0.001 vs. vehicle; left VF (Figure 4E): MSC, 2433 ± 1260; *p* < 0.05 vs. vehicle; left LF (Figure 4F): MSC, 37,855 ± 855; *p* < 0.001 vs. vehicle). These results demonstrate that although MSC infusion did not restore the preserved tracts on the injured side, preserved tract counts on the non-injured side were higher than in the vehicle group, although they remained below intact values. Notably, preserved tracts were also protected in the LF, the region most distant from the lesion, indicating that MSC treatment exerted protective effects even in remote areas of the spinal cord.

Regarding the redirected tracts, only a few redirected tracts were detected on both the injured and non-injured sides in the vehicle group (Figure 4A–C), indicating that spontaneous redirection was minimal under untreated conditions. In contrast, MSC treatment promoted notable redirection, particularly on the non-injured side of the spinal cord. On the injured (right) side, tracts projecting from the right DF at T6 to the contralateral (left) DF at T8 were more frequently observed in the MSC group than in the intact or vehicle groups (Figure 4A). On the non-injured (left) side, redirected tracts projecting from the left DF at T6 to the left LF at T8 were almost absent in the intact group (Figure 4D,G) and sparsely observed in the vehicle group (Figure 4D,H), but were clearly detected in the MSC-treated group (Figure 4D,I) and significantly increased compared with both intact and vehicle groups (215 ± 180 vs. 216 ± 160 vs. 4552 ± 720; *p* < 0.001 for both comparisons; Figure 4D).

Similarly, redirected tracts from the left VF at T6 to the left LF at T8 were almost absent in the intact group (Figure 4E,J) and rarely present in the vehicle group (Figure 4E,K), but were clearly visible in the MSC-treated group (Figure 4E,L) and significantly increased compared with both the intact and vehicle groups (183 ± 183 vs. 52 ± 48 vs. 3409 ± 808; *p* < 0.001; Figure 4E). These results indicate that MSC infusion was preferentially associated with lateral redirection of reconstructed tracts, particularly from dorsal and ventral origins (DF and VF) into the LF on the non-injured side.

### 2.4. MSC Infusion Enhanced Tract Redirection and Preservation After Staggered Lateral Hemisection SCI at the T12 Lesion Level

**Figure 5:**
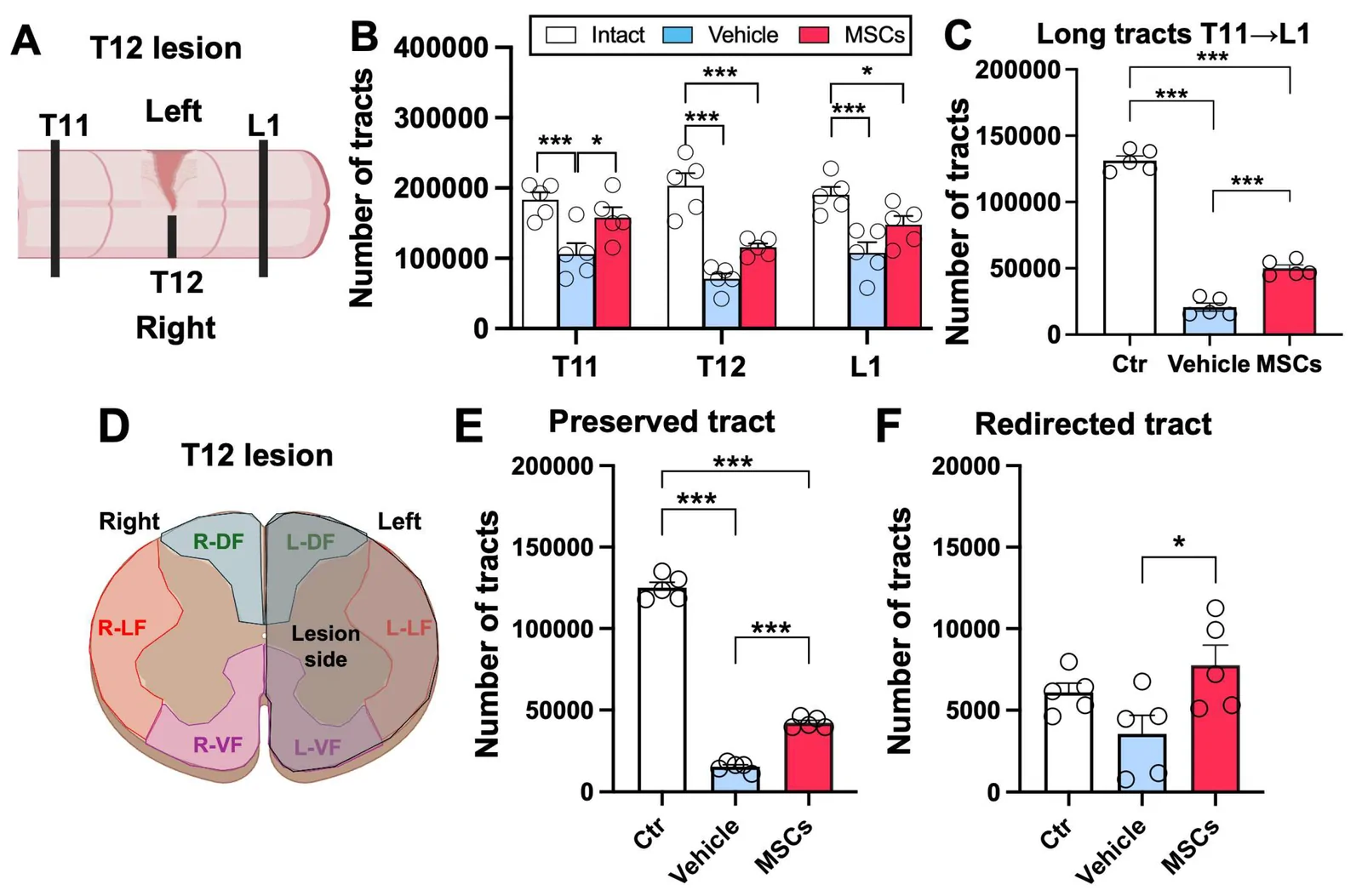
DTI tractography reveals MSC-associated preservation and redirection of reconstructed tracts at the T12 lesion level in staggered lateral hemisection SCI. (**A**) T12 lesion schematic. Schematic of the staggered lateral hemisection SCI model showing a left-sided lesion at T12. Black lines indicate the spinal levels (T11, T12, L1) analyzed by ex vivo DTI. (**B**) Total tract counts. Quantification of tract numbers at T11, T12, and L1 segments. (**C**) Long tract continuity. Quantification of longitudinal tracts spanning from T11 to L1. (**D**) Tract classification. Schematic of spinal cord cross-section at the T12 lesion level indicating the six ROIs: R-DF, L-DF, R-LF, L-LF, R-VF, and L-VF. The lesion side (**left**) is indicated. (**E**) Preserved tracts. (**F**) Redirected tracts. Data are presented as mean ± SEM. Group sizes: Intact (*n* = 5), Vehicle (*n* = 5), MSC (*n* = 5). Statistical analysis: one-way ANOVA with Tukey’s multiple comparisons test. * *p* < 0.05, *** *p* < 0.001. DTI = diffusion tensor imaging, MSC = mesenchymal stem cell, SCI = spinal cord injury, ROI = region of interest, DF = dorsal funiculus, LF = lateral funiculus, VF = ventral funiculus, SEM = standard error of the mean, R = right, L = left.

Next, we examined the reconstructed tract trajectories around the T12 lesion in the staggered lateral hemisection SCI model using DTI tractography (Figure 5A). ROIs were placed to include the entire spinal cord cross-section at T11, T12, and L1, and all tracts traversing each ROI were reconstructed and quantified to evaluate the total tract numbers at each segmental level (Figure 5B). Compared with the intact group, the total number of tracts in the vehicle-treated SCI group was markedly reduced at the T12 lesion level (70,829 ± 7807 vs. 203,304 ± 17,810; *p* < 0.001), indicating substantial tract loss at the primary lesion site. Furthermore, tract numbers were also significantly decreased at both the rostral T11 (105,971 ± 15,745 vs. 183,410 ± 10,702; *p* < 0.001) and caudal L1 (107,698 ± 15,235 vs. 190,455 ± 11,266; *p* < 0.001) levels. In the MSC-treated group, tract numbers at T12 were significantly lower than those in intact controls (115,775 ± 5417 vs. 203,304 ± 17,810; *p* < 0.001) but were significantly higher than those in the vehicle group (70,829 ± 7807; *p* < 0.05). At the rostral T11 level, tract counts in the MSC-treated group (158,157 ± 14,558) were significantly higher than those in the vehicle group (*p* < 0.05) and did not differ significantly from those in intact controls. At the caudal L1 level, MSC tract counts (147,772 ± 12,627) showed a nonsignificant trend toward higher counts (vs. vehicle, *p* = 0.086; vs. Intact, *p* = 0.063). These findings suggest that MSC infusion mitigates the loss of reconstructable tracts caused by T12 hemisection, with tract counts at the lesion core and the rostral T11 level higher than those in the vehicle group.

Subsequently, the longitudinal tracts between T11 and L1 were examined to evaluate long-tract continuity across the lesion around the T12 lesion level (Figure 5C). ROIs were placed at both T11 and L1, and the number of tracts traversing both ROIs was quantified. The analysis revealed that total longitudinal tract numbers spanning from T11 to L1 were markedly reduced in vehicle-treated rats compared with intact controls (20,798 ± 2976 vs. 131,226 ± 3537; *p* < 0.001). In the MSC-treated group, tract counts remained lower than those in the intact group (50,003 ± 2602 vs. 131,226 ± 3537; *p* < 0.001) but were significantly higher than those in the vehicle group (*p* < 0.001), indicating partial restoration of long-tract continuity following MSC infusion (Figure 5C).

Next, we analyzed the trajectory patterns of the tracts across the lesion at the T12 lesion level. As illustrated in Figure 5D, six ROIs were placed across the cross-sectional area of the spinal cord at T11 and L1, corresponding to the left and right DF, LF, and VF funiculi. Six ROIs were defined at each level. Each tract passing through a given ROI at T11 was traced to determine which of the six ROIs at L1 it entered. All 36 possible tract trajectories derived from the 6 (T11) × 6 (L1) ROI combinations were quantitatively analyzed and classified into two categories, “Preserved tract” and “Redirected tract,” as shown in Figure 3E. Quantitative analysis revealed that preserved tracts were markedly reduced in the vehicle group compared with intact controls (15,349 ± 1259 vs. 125,127 ± 3343; *p* < 0.001, Figure 5E). In the MSC-treated group, the number of preserved tracts (42,244 ± 1430) was lower than that in intact controls (*p* < 0.001) but was significantly higher than that in the vehicle group (*p* < 0.0001), demonstrating partial preservation of longitudinal tract continuity across the T12 lesion (Figure 5E). Considering redirected tracts (Figure 5F), the vehicle group (3559 ± 1135) had slightly lower redirected tracts than those in intact controls (6099 ± 565); MSC infusion significantly increased redirected tracts compared with the vehicle group (7759 ± 1229; *p* < 0.05 vs. vehicle), although not significantly different from those in intact controls. Taken together, preserved tract counts were higher in the MSC group than in the vehicle group, although they remained below intact values. Redirected tract counts were also higher in the MSC group than in the vehicle group, although at T12 they did not differ significantly from intact values.

### 2.5. MSC Infusion Preferentially Promotes Redirected Tracts Across Specific Pathways Around the T12 Lesion Level

**Figure 6:**
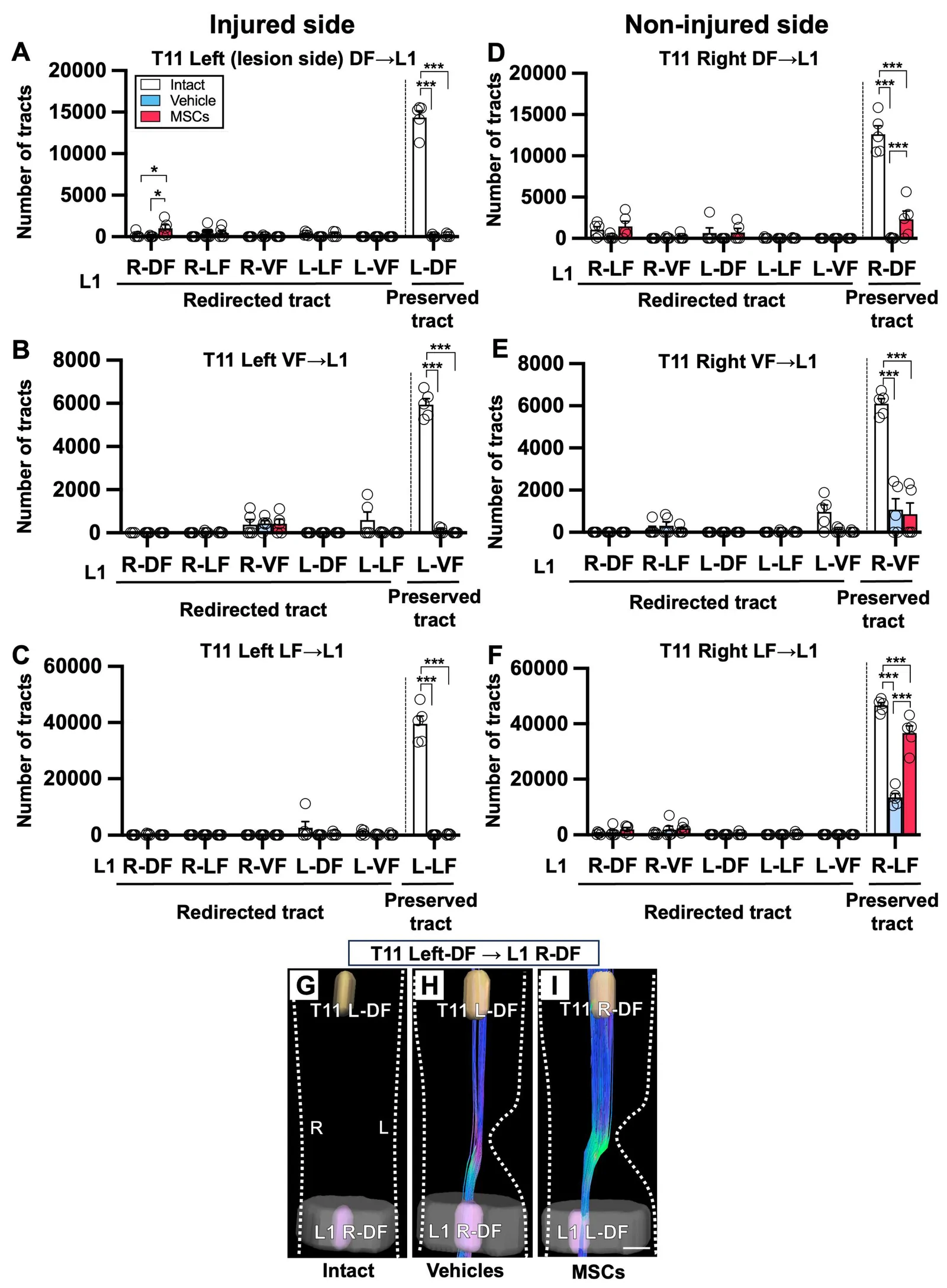
MSC infusion preferentially promotes redirected tracts across specific pathways at the T12 lesion level. (**A**–**C**) Quantification of tracts originating from the injured (**left**) side at T11. (**A**) Left DF at T11 (L-DF → L1). (**B**) Left VF at T11 (L-VF → L1). (**C**) Left LF at T11 (L-LF→L1). (**D**–**F**) Quantification of tracts originating from the non-injured side (**right**) at T11. (**D**) Right DF at T11 (R-DF → L1). (**E**) Right VF at T11 (R-VF → L1). (**F**) Right LF at T11 (R-LF → L1). (**G**–**I**) Representative DTI tractography images showing redirected tracts from T11 L-DF to L1 R-DF (midline crossing). (**G**) Intact controls show no evident midline crossing. (**H**) Vehicle-treated rats display some midline-crossing tracts. (**I**) MSC-treated rats exhibit markedly increased L-DF to R-DF midline crossing. Scale bar = 500 μm. Data are presented as mean ± SEM. Group sizes: Intact (*n* = 5), Vehicle (*n* = 5), MSC (*n* = 5). Statistical analysis: one-way ANOVA with Tukey’s multiple comparisons test. * *p* < 0.05, *** *p* < 0.001. DTI = diffusion tensor imaging, MSC = mesenchymal stem cell, DF = dorsal funiculus, LF = lateral funiculus, VF = ventral funiculus, SEM = standard error of the mean, R = right, L = left, ANOVA = analysis of variance.

To determine which tracts were preserved or redirected around the T12 lesion, we traced the longitudinal tracts passing through each of the six ROIs at T11 to their corresponding ROIs at L1 using DTI tractography (Figure 5D). The tracts running through the injured side (left), including the left DF, VF, and LF at T11, are shown in Figure 6A–C, respectively, whereas those running through the non-injured side (right) are shown in Figure 6D–F. In the vehicle group, preserved tracts passing through DF (Figure 6A), VF (Figure 6B), and LF (Figure 6C) on the lesioned (left) side were markedly reduced compared to intact controls. Even on the non-injured side (right), the preserved tracts in the DF (Figure 6D), VF (Figure 6E), and LF (Figure 6F) were significantly reduced in the vehicle group compared with intact controls. These findings suggest that axonal degeneration extended beyond the lesioned side of the spinal cord, affecting the funiculi adjacent to the lesion (right DF and VF), and the most distant region (right LF) on the non-injured side.

In contrast, the preserved tracts on the injured (left) side, including the left DF (Figure 6A), VF (Figure 6B), and LF (Figure 6C), were reduced in the MSC-treated group compared with intact controls, similar to the reductions observed in the vehicle group. Notably, even on the injured side (left), LF (Figure 6C), where the tracts had been redirected (Figure 4D,E) and preserved (Figure 4F) around the T7 lesion level, the number of preserved tracts markedly decreased at the T12 lesion level (Figure 6C). On the non-injured side (right), preserved DF (Figure 6D) and LF (Figure 6F) tract numbers were significantly higher in the MSC-treated group than in the vehicle group, but lower than that in intact controls (Right DF: Intact, 12,631 ± 1020; Vehicle, 22 ± 22; MSC, 2303 ± 1003; *p* < 0.01, MSC vs. Vehicle; Right LF: Intact, 46,338 ± 847; Vehicle, 13,419 ± 1388; MSC, 36,675 ± 2588; *p* < 0.001, MSC vs. Vehicle).

Regarding the redirected tracts around T12, only a few redirected tracts were detected on both the lesioned and non-injured sides in the intact and vehicle groups (Figure 6A–H), indicating minimal spontaneous redirection under untreated conditions. In contrast, MSC treatment promoted notable redirection. On the lesioned (left) side, tracts projecting from the left DF (Figure 6A) at T11 to the contralateral (right) DF at L1 were clearly visible (Figure 6A,I) and were significantly increased compared with both intact and vehicle groups (Intact, 185 ± 170; Vehicle, 47 ± 36; MSC, 1047 ± 409; *p* <0.05 vs. Intact; *p* < 0.05 vs. vehicle; Figure 6A,G–I). On the non-injured side (right), tracts redirecting from the right DF (Figure 6D) to the right LF were more frequent in the MSC group than in the vehicle group, although the difference was not significant. At the T7 lesion level, the number of tracts redirected from the non-injured side of DF to LF (Figure 4D) was significantly increased in the MSC-treated group; this difference was not obvious at the T12 lesion level (Figure 6D). In contrast, the VF-to-LF redirection (Figure 6E), which was markedly enhanced by MSC infusion at the T7 lesion level (Figure 4E), was not observed at the T12 lesion level. Collectively, these findings indicate that at the T12 lesion level, the non-injured side of LF, which had sustained significant loss due to the rostral T7 lesion, did not participate in the redirection patterns that were prominent at T7. Thus, compensatory reorganization at T12 occurred primarily through a DF-mediated pathway, in which tracts from the lesioned side were redirected across the midline into the contralateral DF in this model.

## 3. Discussion

**Figure 7:**
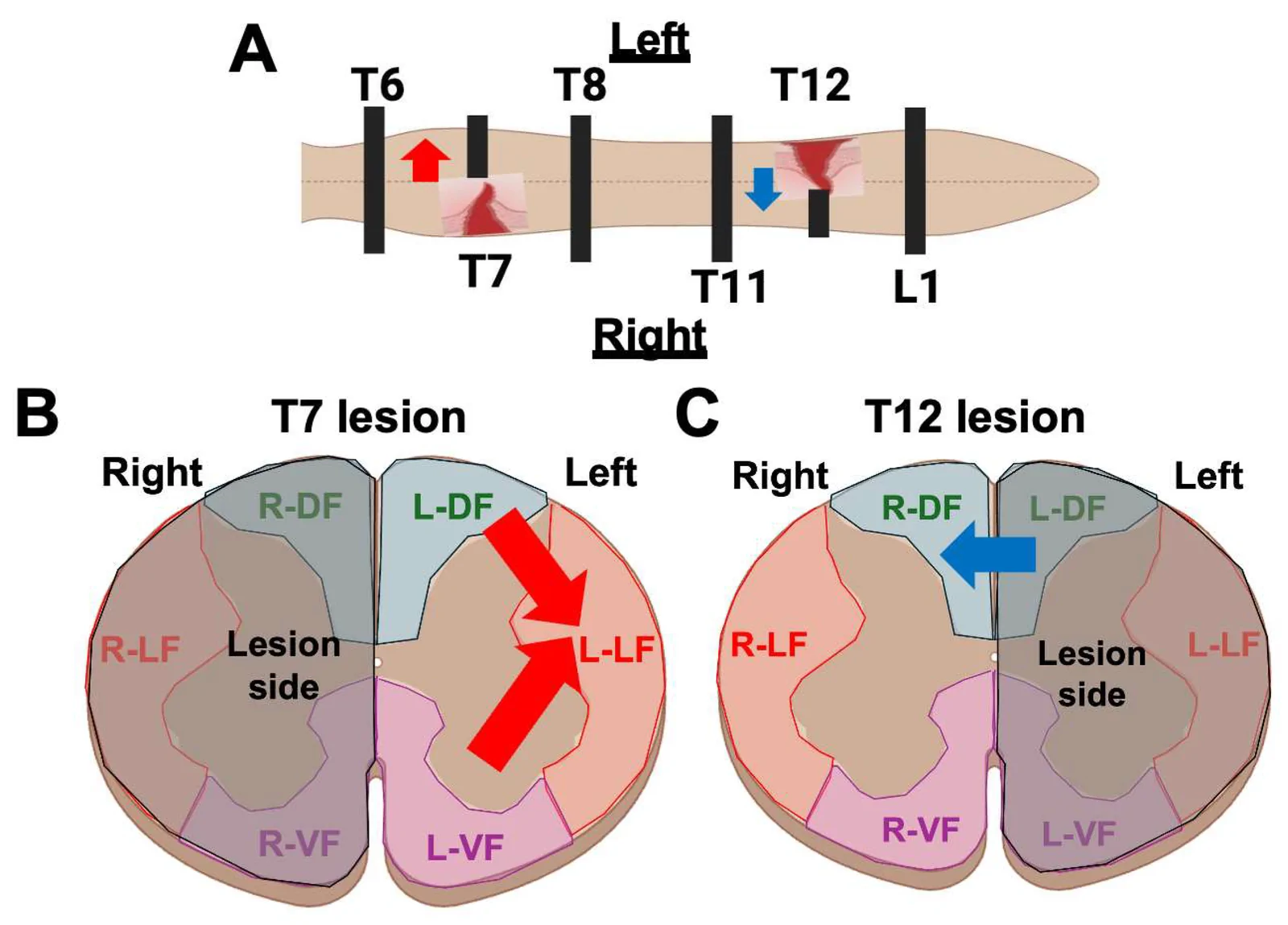
Schematic illustration of MSC-induced tract reorganization patterns in the staggered lateral hemisection SCI model. (**A**) Overview of the staggered lateral hemisection SCI model showing a right-sided lesion at T7 and a left-sided lesion at T12. Black bars indicate the spinal levels analyzed using ex vivo DTI (T6, T7, T8 for the rostral lesion; T11, T12, L1 for the caudal lesion). Red arrow indicates the T7 lesion level; blue arrow indicates the T12 lesion level. (**B**) Cross-sectional schematic at the T7 lesion level (right-sided lesion). Red arrows indicate major redirection patterns observed following MSC infusion: spared tracts in the non-injured side (**left**) DF and VF were redirected laterally into the left LF, forming a lateral detour pathway bypassing the lesion core. (**C**) Cross-sectional schematic at the T12 lesion level (left-sided lesion). Blue arrow indicates the redirection pattern observed following MSC infusion: tracts from the injured side (**left**) DF were redirected across the midline into the contralateral (**right**) DF, forming a compensatory interhemispheric pathway. Unlike the LF-mediated lateral redirection prominent at T7, the T12 lesion level exhibited DF-to-DF midline crossing as the primary compensatory route. MSC = mesenchymal stem cell; SCI = spinal cord injury; DTI = diffusion tensor imaging; DF = dorsal funiculus; LF = lateral funiculus; VF = ventral funiculus.

In the current study, functional recovery was observed after IV infusion of MSCs in a staggered lateral hemisection rat model of SCI. This model, which creates anatomically separated hemisections at the right T7 and left T12, enables a detailed examination of axonal preservation and compensatory plasticity across distant lesion sites. Using this system, we identified distinct MSC-induced patterns of tract redirection at each lesion level (Figure 7). Ex vivo DTI analyses revealed that spared axonal tracts in the DF and VF of the non-injured side at the T7 lesion level were redirected laterally into the LF, thereby bypassing the lesion (Figure 7A,B). In contrast, at the T12 lesion, the LF-mediated lateral pathway was not involved in lesion bypass. The longitudinal tracts running through the DF on the injured side were redirected across the midline into the contralateral DF (Figure 7A,C), indicating the recruitment of a different compensatory route at the caudal lesion level.

### 3.1. DF-to-LF Redirection at the T7 Lesion Level Following IV-MSC Treatment

The spared long tracts running through the DF of the non-injured side at the T7 lesion were preferentially redirected into the LF following MSC infusion compared with vehicle treatment (Figure 7A,B). The observed DF-to-LF redirection is consistent with previous reports describing CST axonal reorganization following SCI. In rodents, the dorsal CST (dCST), which comprises approximately 95% of the descending CST axons, descends within the DF [12]. Following SCI, dCST axons undergo structural reorganization, which enables bypassing of the lesion site via multiple mechanisms. First, dCST axons establish new contacts with long descending propriospinal neurons, whose axons course through the LF to bypass the lesion and relay cortical motor commands to the lumbar motor circuits [13,14]. Second, direct evidence demonstrates that dCST axons form bridging connections with axons in the LF, with fine-caliber axons projecting radially from the dCST into the LF and converging back to the dCST caudal to the lesion, forming a detour pathway around the injury site [6]. Previous research using adeno-associated virus (AAV)-based anterograde tracing in a contusion SCI model further showed that IV MSC therapy enhances these bridging communications between the dCST and axons in the LF, both rostral and caudal to the lesion, suggesting that MSCs facilitate collateral sprouting or enhancement of pre-existing minor CST fibers [6]. These findings support the notion that the DF-to-LF redirection observed in our DTI analysis reflected both spontaneous and MSC-enhanced corticospinal pathway reorganization.

### 3.2. VF-to-LF Redirection at T7 Lesion Level Following IV-MSC Treatment

In addition to the DF-to-LF reorganization, DTI revealed that the spared long tracts coursing through the non-injured side of the VF were also preferentially redirected into the LF following MSC infusion (Figure 7A,B). In contrast, direct anatomical evidence demonstrating the trajectory change in VF tracts to course through the LF after SCI remains lacking, despite extensive research on spinal cord plasticity. However, numerous studies have suggested functional linkages between the VF and LF systems. The ventral CST (vCST) in the VF, although numerically minor compared to dCST, can undergo compensatory sprouting after dorsal pathway damage, expanding into spinal regions originally dominated by the LF pathways [12,15]. Similarly, the reticulospinal tract (ReST), which descends in the VF, works coordinately with corticospinal pathways in the LF to regulate locomotor output. When CST function in the LF is compromised following SCI, the ReST plays a more prominent role in motor control, effectively compensating for LF-mediated functions [16]. These observations suggest that the VF and LF operate as coordinated systems after SCI; however, the structural basis of this coordination remains unclear. Similar to the DF-to-LF reorganization described above, wherein fine-caliber pre-existing axons are enhanced (increased axonal diameter) after MSC treatment, rendering them more readily detectable [6], the MSC infusion-induced VF-to-LF redirection detected in the present study using ex vivo DTI may reflect a similar mechanism. Fine-caliber fibers connecting the VF and LF systems may undergo MSC-induced enhancement, with an increase in their diameter enabling the detection of trajectory changes by DTI analysis, thereby revealing this previously unrecognized structural relationship.

### 3.3. MSC-Induced DF Redirection Across the Midline into the Contralateral DF at the T12 Lesion Level

At the T12 lesion level, a redirectional pattern, distinct from that observed at T7, emerged following MSC infusion. Although lateral detouring through the LF was a prominent bypass route at the T7 lesion level, supported by the reorganization of the VF–LF and DF–LF pathways, this LF-mediated pathway was not obvious at T12. Instead, the spared long tracts running through the injured DF were redirected across the midline into the contralateral non-injured side of DF at the T12 lesion level (Figure 7A,C). This midline crossing CST redirection has also been described as a compensatory mechanism during spontaneous recovery after spinal hemisection [17] and contusion [6]. MSC therapy may reinforce this pathway by promoting collateral sprouting or enhancing pre-existing minor CST fibers, similar to enhanced corticospinal networks reported after IV-MSC infusion [6]. These observations are consistent with the DF-mediated midline crossing redirection detected using DTI at the T12 level in the present study.

At the T7 lesion level, the number of redirected tracts from the non-injured side (left) of the DF or VF to the LF (Figure 7A,B) was significantly increased in the MSC-treated group; this difference was not obvious at the T12 lesion level (Figure 7A,C). The distinct redirection patterns observed at the T7 and T12 lesion levels can be explained by a reduction in axonal capacity for redirection at the caudal lesion site. At the T12 lesion level, the non-injured side (right) of the DF and VF had already substantially lost long tracts due to the rostral T7 lesion on the right side, leaving insufficient axonal substrates to support redirection into the LF. Consequently, only the DF of the injured side (left) at T12, which remained intact after the T7 lesion, participated in compensatory reorganization, resulting in the observed DF-to-DF midline crossing pattern. This finding demonstrates that when redirection occurs at one spinal level, the same redirection pattern cannot be readily established at a second lesion. The limited capacity for adaptive redirection across multiple lesion sites highlights a critical limitation in neural plasticity following multiple SCIs.

### 3.4. Advantages of DTI Tractography in Evaluating Axonal Reorganization After SCI

DTI tractography is uniquely advantageous for assessing the organization of axonal pathways in the injured spinal cord [18]. By defining ROIs at multiple segmental levels, DTI reconstructs long tracts traversing these regions, allowing a comprehensive evaluation of spared and redirected fibers [19,20]. Unlike conventional histological methods, such as viral vector–based anterograde or retrograde tracing, which label only the neurons targeted by the injection or genetic strategy [21,22], DTI can visualize the entire three-dimensional axonal architecture, including the corticospinal, rubrospinal, and reticulospinal systems, without assumptions regarding their origins or terminations. Our previous study, using a contusive SCI model, showed that the injured dCST developed an enhanced axonal network running parallel to minor CST components within the spared spinal tissue following IV MSC infusion [6]. Although histological tracer studies can confirm these findings, they remain limited to well-characterized pathways, such as the CST [6] and RST [7]. In contrast, ex vivo DTI enables quantitative analysis of axonal trajectories throughout the spinal cord or within any chosen region [23,24,25]. By detecting anisotropic water diffusion along axonal fibers, ex vivo DTI allows comprehensive assessment of all tracts throughout the spinal cord while also enabling focused analysis of specific regions or pathways of interest. This dual capability provides both global and region-specific views of structural plasticity. Importantly, by adjusting the spatial separation between ROIs, DTI tractography can be configured to selectively reconstruct long-distance projecting fibers while excluding short-range tracts, thereby minimizing the contributions from local interneuronal circuits. This capacity to preferentially analyze long descending pathways allows the isolation of reorganized long tracts most relevant for functional recovery, while effectively filtering out short propriospinal or interneuron-dominated connections. This advantage is particularly valuable for characterizing MSC-induced network reorganization in which multiple descending systems may interact to establish alternative, functionally compensatory routes around the lesion. Overall, DTI tractography offers an integrative network-level view of axonal remodeling that complements histological methods and is indispensable for elucidating recovery mechanisms following MSC therapy.

### 3.5. The Therapeutic Potential of IV-MSC Infusion for Multiple SCIs

SCI with damage at two sites, referred to as “NDSI,” presents a complex clinical challenge and is often associated with severe neurological deficits [1,2] and a prolonged, complicated rehabilitation course [26]. The pathophysiologies underlying these outcomes involve multiple factors. However, our findings provide a potential mechanistic explanation for these distinct outcomes; they revealed that MSC-induced neural longitudinal tract redirection differed distinctly between the T7 and T12 levels. Specifically, tract redirection into the non-injured LF, predominant around the T7 lesion, was compromised in the caudal T12 lesion. This finding indicates that the intrinsic capacity of the spared axons to undergo adaptive redirection is limited and becomes substantially impaired when multiple lesions coexist along the spinal axis. Consequently, the presence of a second lesion probably inhibits the compensatory recovery pathways that operate in single-site injuries, thereby causing failure of spontaneous motor function recovery. This study confirmed the therapeutic efficacy of IV MSC treatment, even in a double-lesion model, and showed that redirected tracts were more frequent in the MSC group than in the vehicle group at both lesion levels.

### 3.6. Limitations

Several limitations should be considered when interpreting these results. First, a reconstructed tract is a trajectory computed from the voxel-wise diffusion tensor field, not of individual axons. Tract counts are relative, semi-quantitative measurements obtained using the pipeline described in Section 4.5, and tractography cannot establish the axonal identity of these trajectories or exclude reconstruction artifacts. Therefore, a redirected tract indicates a change in the reconstructed trajectory and is not confirmed by histological analyses. Second, the structural analysis was performed at a single time point (4 weeks), and only male rats were studied. Finally, the present data indicate that intravenous infusion of MSCs improved locomotor function and altered reconstructed tract patterns. It remains unknown how the patterns and extent of these alterations contribute to locomotor recovery.

## 4. Materials and Methods

All animal procedures were performed in accordance with the National Institutes of Health guidelines for the care and use of laboratory animals and the institutional guidelines of Sapporo Medical University, and were approved by the Institutional Animal Care and Use Committee and the Institutional Biosafety Committee for Recombinant DNA Research of Sapporo Medical University.

### 4.1. Preparation of MSCs from Rat Bone Marrow

MSCs were prepared and cultured according to our previous studies [27,28,29]. Briefly, bone marrow obtained from the femoral bones of 5 adult (6–8 weeks old) Sprague-Dawley (SD) rats was diluted to 15 mL with Dulbecco’s modified Eagle’s medium (DMEM) (Sigma, St. Louis, MO, USA) supplemented with 10% heat-inactivated fetal bovine serum (Thermo Fisher Scientific Inc., Waltham, MA, USA), 2 mM L-glutamine (Sigma), 100 U/mL penicillin, and 0.1 mg/mL streptomycin (Thermo Fisher Scientific Inc.), and incubated for 3 days at 37 °C in a humidified atmosphere containing 5% CO_2_. When cultures almost reached confluence, the adherent cells were detached with 0.25% trypsin–ethylenediaminetetraacetic acid solution (Sigma) and subcultured at 1 × 10^4^ cells/mL of medium. MSCs obtained after three passages were used in the present study. MSCs prepared using this protocol show a surface antigen profile of CD45−, CD73+, CD90+, and CD106− by flow cytometric analysis [27,30]. They also give rise to mesenchymal derivatives, namely osteocytes, adipocytes, and chondrocytes, after culture in osteogenic, adipogenic, and chondrogenic induction media, as demonstrated by Alizarin red S, Oil red O, and Alcian blue staining, respectively [31].

### 4.2. Staggered Lateral Hemisection SCI Model

A staggered lateral hemisection SCI model, at the right T7 and left T12 levels, was used [11,14] (Figure 1A–C). Adult male SD rats aged 8 weeks (250–300 g) were anesthetized using an intraperitoneal injection of ketamine (90 mg/kg) and xylazine (4 mg/kg) [6,32]. The initial lesion was induced at the left T12 level. Following the incision, the left T11/12 facet joint was exposed, the inferior articular process of the left T11 was removed, and part of the superior articular process of the left T12 was resected. This procedure allowed the exposure of the spinal cord from the midline to the left lateral margin. Subsequently, the right T6/7 facet joint was exposed, the inferior articular process of the right T6 was removed, and part of the superior articular process of the right T7 was resected, thereby exposing the spinal cord from the midline to the right lateral margin. The right half of the spinal cord at the T7 level (Figure 1B) and the left half at the T12 level (Figure 1C) were then hemisected using a microblade for ophthalmic surgery (p-715, Feather Co., Ltd., Osaka, Japan). Postoperative care included manual bladder expression twice daily for 14 days. Rats were housed under controlled environmental conditions (50% humidity, 24 ± 2 °C). SCI rats were randomized to a vehicle group (*n* = 5) or an MSC group (*n* = 5), and were assessed using the BBB locomotor rating scale over 4 weeks and subsequently with ex vivo DTI; 5 non-SCI rats served as intact controls.

### 4.3. Behavioral Testing

Open-field locomotor function was assessed using the BBB locomotor rating scale [33]. The BBB score consists of a combination of hind limb movements, trunk position and stability, stepping, coordination, paw placement, toe clearance, and tail position [33]. In this study, the weekly BBB test was strictly based on objective criteria as mentioned in previous studies; the animals were included only if they had BBB scores of 0 on day 1 post SCI [6,7,34]. The BBB score was assessed by a tester blinded to the treatment [35,36]. The rats were scored on days 0, 1, 3, 7, 14, 21, and 28 post SCI (*n* = 5 per group).

### 4.4. Experimental Protocols

The experimental protocol is illustrated in Figure 1. SCI was induced in SD rats and were randomized into two experimental groups as follows: (1) the rats that were injected intravenously with vehicle only (vehicle group [*n* = 5], 1 mL fresh DMEM, no cells) on day 1 after SCI induction; (2) the rats injected intravenously with an infusion of MSCs (MSC group [*n* = 5], 1 × 10^6^ cells each) in 1 mL of fresh DMEM on day 1 after SCI induction. An IV infusion was administered via the femoral veins [6,10,29]. After the initial MSC or vehicle infusion, follow-up was performed for 4 weeks. The remaining cells were immediately tested for high viability (>99%) using 0.4% trypan blue. The number of MSCs used for each injection was determined based on previous studies [6,10,36,37]. Behavioral testing was conducted on postoperative days 0, 1, 3, 7, 14, 21, and 28. As this study did not involve autologous transplantation, we used cyclosporine A to avoid potential immune reactions. All rats were subcutaneously injected daily with cyclosporine A (10 mg/kg) from the day before MSC or vehicle infusion on day 1 after SCI induction [6,27,34]. After the animals were euthanized, ex vivo DTI analyses were performed.

### 4.5. Ex Vivo MRI DTI Studies

Ex vivo MRI DTI studies were performed using methods (including the anesthesia protocol) described in our previous studies [10,29,38]. Rats were deeply anesthetized with an intraperitoneal injection of ketamine (50 mg/kg) and xylazine (10 mg/kg), followed by perfusion with phosphate-buffered saline (PBS) and 4% paraformaldehyde (PFA), and harvesting of the spinal cords. The spinal cords were stored in 4% PFA at 4 °C for 2 weeks. The fixed spinal cords were dissected into 2 cm blocks at the T7 and T12 levels and soaked in a fluorine solution to minimize susceptibility artifacts at the interface [10,39]. DTIs were obtained using a 7.0 T MRI scanner (70/16 PharmaScan, Bruker Biospin MRI GmbH, Ettlingen, Germany): b-value = 800 s/mm^2^, diffusion encoding directions = 30, number of signal averages = 10, TR = 1300.0 ms, TE = 18.0 ms, slice thickness = 0.5 mm without gap, matrix size 112.5 × 200.0 μm, 55 slices, and total scan time = approximately 9 h.

Deterministic streamline tractography was performed using DSI Studio (Chen build, 17 May 2024; https://dsi-studio.labsolver.org, accessed on 17 May 2024) with the algorithm developed by Yeh et al. [40]. FA was used as the tracking index. Tracking parameters were determined in preliminary analyses, applied without further modification, and based on previous DSI Studio tractography studies [41,42,43]. An anisotropy threshold of 0 invoked parameter saturation [44], in which the threshold for each seed was randomly selected between 0.5 and 0.7 of Otsu’s threshold. The angular threshold was set to 90 degrees, as previously used for spinal cord tractography [45]. The step size was set to the voxel spacing (0.1125 × 0.1125 × 0.5 mm). Tracts longer than 40 mm were discarded, and 1,000,000 seeds were placed in each region of interest. ROIs for fiber tracking were placed by an operator blinded to treatment, defined on each axial image of the spinal cord according to the anatomical landmarks at the T7 and T12 lesion levels (Figure 1A). For each lesion level, the ROIs were placed at three segmental positions: the lesion epicenter, one segment rostral, and one segment caudal to the epicenter. In the T7 lesion model, ROIs were positioned at T6 (rostral, 2.5 mm rostral to the lesion epicenter), T7 (lesion epicenter), and T8 (caudal, 2.5 mm caudal to the lesion epicenter), as shown in Figure 3A. In the T12 lesion model, the ROIs were defined at the T11 (rostral), T12 (lesion epicenter), and L1 (caudal) levels (Figure 5A). To evaluate changes in long-tract trajectories across the lesion, six ROIs were delineated at the rostral and caudal levels corresponding to the L-DF, R-DF, L-LF, R-LF, L-VF, and R-VF (Figure 3D and Figure 5D) in line with previous studies [46,47,48]. Identical acquisition and tracking parameters were used for all specimens. Tract counts are relative, semi-quantitative measurements and do not represent absolute numbers of axons.

A reconstructed tract (streamline) represents a computed three-dimensional trajectory that follows the principal diffusion direction from one voxel to another, depicting the inferred course of a coherently oriented fiber bundle rather than an individual axon. Accordingly, the number of streamlines passing through a given ROI is referred to as the tract count. Tract counts were therefore directly comparable across the intact, vehicle, and MSC groups, with between-group differences reflecting the relative changes in reconstructable tract continuity. Reconstructed tracts were classified into two major categories: redirected and preserved tracts (Figure 3E). Redirected tracts were defined as reconstructed tracts entering a different anatomical region at the caudal level compared with their rostral origin (for example, a tract originating in the L-DF at T6 that shifted into the L-LF at T8; Figure 3E, right). In contrast, preserved tracts were defined as reconstructed tracts that maintained their original trajectory across the corresponding segmental levels (e.g., L-DF at T6 continued to L-DF at T8; Figure 3E, left). Throughout this study, preserved and redirected tracts are operational categories defined by the regions of interest through which a reconstructed tract passes at the rostral and caudal levels. These terms describe the trajectory of a reconstructed tract but do not establish the axonal identity.

### 4.6. Statistical Analysis

All statistical analyses were performed using GraphPad Prism software (version 9.0; GraphPad Software, San Diego, CA, USA). Repeated-measures two-way analysis of variance (ANOVA) followed by Šidák post hoc testing were used for multiple comparisons of BBB scores. For other continuous variables, the Shapiro–Wilk test was used to assess the normality of the data distribution. Data that satisfied the assumption of normality were analyzed using one-way ANOVA, followed by the Tukey–Kramer post hoc test for multiple comparisons. Data that did not satisfy the assumption of normality were analyzed using the Kruskal–Wallis test, followed by the Steel–Dwass post hoc test. Data are presented as mean ± SEM. All experimental procedures and data reporting conformed to the Animals in Research: Reporting in Vivo Experiments (ARRIVE) guidelines [49] and the Minimum Information about a Spinal Cord Injury Experiment (MIASCI) standard [50].

## 5. Conclusions

This study demonstrated that intravenous infusion of MSCs improved locomotor outcome and altered the patterns of spinal cord tract trajectories reconstructed from DTI data in a rat staggered lateral hemisection model of SCI. Ex vivo DTI revealed characteristic dorsal-to-lateral, ventral-to-lateral, and midline-crossing patterns, with differences between the two lesion levels. DTI-based tractography provides an overview of these reconstructed tract patterns.

## Data Availability

The original contributions presented in this study are included in the article. Further inquiries can be directed to the corresponding author.
